# Evidence of escape of SARS-CoV-2 variant B.1.351 from natural and vaccine-induced sera

**DOI:** 10.1016/j.cell.2021.02.037

**Published:** 2021-04-29

**Authors:** Daming Zhou, Wanwisa Dejnirattisai, Piyada Supasa, Chang Liu, Alexander J. Mentzer, Helen M. Ginn, Yuguang Zhao, Helen M.E. Duyvesteyn, Aekkachai Tuekprakhon, Rungtiwa Nutalai, Beibei Wang, Guido C. Paesen, Cesar Lopez-Camacho, Jose Slon-Campos, Bassam Hallis, Naomi Coombes, Kevin Bewley, Sue Charlton, Thomas S. Walter, Donal Skelly, Sheila F. Lumley, Christina Dold, Robert Levin, Tao Dong, Andrew J. Pollard, Julian C. Knight, Derrick Crook, Teresa Lambe, Elizabeth Clutterbuck, Sagida Bibi, Amy Flaxman, Mustapha Bittaye, Sandra Belij-Rammerstorfer, Sarah Gilbert, William James, Miles W. Carroll, Paul Klenerman, Eleanor Barnes, Susanna J. Dunachie, Elizabeth E. Fry, Juthathip Mongkolsapaya, Jingshan Ren, David I. Stuart, Gavin R. Screaton

**Affiliations:** 1 Division of Structural Biology, Nuffield Department of Medicine, University of Oxford, The Wellcome Centre for Human Genetics, Oxford, UK; 2 Wellcome Centre for Human Genetics, Nuffield Department of Medicine, University of Oxford, Oxford, UK; 3 Chinese Academy of Medical Science (CAMS), Oxford Institute (COI), University of Oxford, Oxford, UK; 4 Oxford University Hospitals NHS Foundation Trust, Oxford, UK; 5 National Infection Service, Public Health England (PHE), Porton Down, Salisbury, UK; 6 Peter Medawar Building for Pathogen Research, Oxford, UK; 7 Nuffield Department of Clinical Neurosciences, University of Oxford, Oxford, UK; 8 Nuffield Department of Medicine, University of Oxford, Oxford, UK; 9 NIHR Oxford Biomedical Research Centre, Oxford, UK; 10 Oxford Vaccine Group, Department of Paediatrics, University of Oxford, Oxford, UK; 11 Worthing Hospital, Worthing, UK; 12 MRC Human Immunology Unit, MRC Weatherall Institute of Molecular Medicine, Radcliffe Department of Medicine, University of Oxford, Oxford, UK; 13 Jenner Institute, Nuffield Department of Medicine, University of Oxford, Oxford, UK; 14 Sir William Dunn School of Pathology University of Oxford, Oxford, UK; 15 Translational Gastroenterology Unit, University of Oxford, Oxford, UK; 16 Centre For Tropical Medicine and Global Health, Nuffield Department of Medicine, University of Oxford, Oxford, UK; 17 Mahidol-Oxford Tropical Medicine Research Unit, Bangkok, Thailand; 18 Siriraj Center of Research Excellence in Dengue & Emerging Pathogens, Dean Office for Research, Faculty of Medicine Siriraj Hospital, Mahidol University, Thailand; 19 Diamond Light Source Ltd, Harwell Science & Innovation Campus, Didcot, UK; 20 Instruct-ERIC, Oxford House, Parkway Court, John Smith Drive, Oxford, UK

**Keywords:** SARS-CoV-2, variant, B.1.351, neutralization, escape, antibody, vaccine, South Africa, receptor-binding domain, ACE2

## Abstract

The race to produce vaccines against severe acute respiratory syndrome coronavirus 2 (SARS-CoV-2) began when the first sequence was published, and this forms the basis for vaccines currently deployed globally. Independent lineages of SARS-CoV-2 have recently been reported: UK, B.1.1.7; South Africa, B.1.351; and Brazil, P.1. These variants have multiple changes in the immunodominant spike protein that facilitates viral cell entry via the angiotensin-converting enzyme-2 (ACE2) receptor. Mutations in the receptor recognition site on the spike are of great concern for their potential for immune escape. Here, we describe a structure-function analysis of B.1.351 using a large cohort of convalescent and vaccinee serum samples. The receptor-binding domain mutations provide tighter ACE2 binding and widespread escape from monoclonal antibody neutralization largely driven by E484K, although K417N and N501Y act together against some important antibody classes. In a number of cases, it would appear that convalescent and some vaccine serum offers limited protection against this variant.

## Highlights

• Reduced B.1.351 neutralization by mAbs and sera induced by early SARS-CoV-2 isolates
• B.1.351 neutralization titer reduced 8- to 9-fold for Pfizer and AstraZeneca vaccinees
• E484K, K417N, and N501Y cause widespread escape from mAbs
• NTD deletion in B.1.351 abrogates neutralization by a potent neutralizing human mAb

## Graphical abstract

**Figure:**
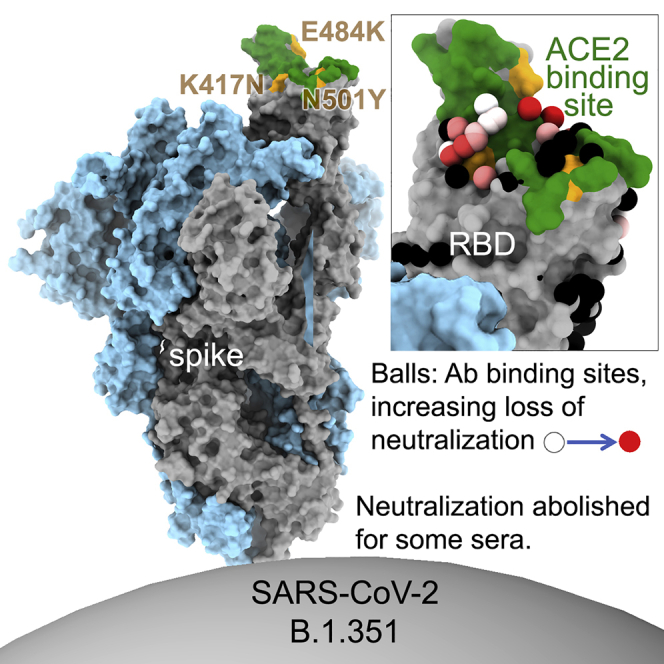

## Teaser

Structure-function analysis of the SARS-CoV-2 variant B.1.351 using serum samples from convalescent and vaccinated individuals reveals how mutations in the viral spike protein result in tighter binding to the receptor ACE2 and allow escape from monoclonal antibody neutralization.

## Introduction

Reports of a severe acute respiratory syndrome (SARS) emerged in December 2019, with rapidly increasing cases and deaths in Wuhan, China. The virus, SARS-coronavirus 2 (CoV-2) was rapidly identified, with the sequence published in January 2020 (Lu et al., 2020), and the disease it caused subsequently named coronavirus disease 2019 (COVID-19). SARS-CoV-2 has been estimated to have infected at least 118 million people, with 2.6 million deaths worldwide (https://www.worldometers.info/coronavirus).

An unprecedented global scientific effort has been led by multiple pharmaceutical companies and academic laboratories to produce vaccines against SARS-CoV-2 (https://www.who.int/publications/m/item/draft-landscape-of-covid-19-candidate-vaccines), seen by many as the only realistic way to release populations from the harsh social isolation measures being implemented in many countries and the consequential severe economic disruption (Keogh-Brown et al., 2020). Many vaccine candidates have been developed, all aiming to generate antibody (and T cell) responses against the spike protein of SARS-CoV-2. These have been developed using spike sequences derived from the early Wuhan strain and include recombinant protein, inactivated virus, RNA, and virally vectored platforms (Krammer, 2020). With accelerated trials, the first efficacy results were delivered around 10 months following the first publication of the sequence of SARS-CoV-2 (Polack et al., 2020; Voysey et al., 2021a; Baden et al., 2021).

Impressive results have now been reported from a variety of manufacturers: Novavax-recombinant spike and Janssen-adenoviral-vectored vaccines have recently reported good efficacy (https://www.novavax.com/sites/default/files/2021-02/20210202-NYAS-Novavax-Final.pdf; https://www.medscape.com/viewarticle/944933), while Moderna-RNA, Pfizer/BioNTech-RNA, and Oxford-AstraZeneca-chimp adenoviral-vectored vaccines have already received emergency use authorization (EUA) in a number of countries and will be deployed at massive scale in 2021.

In the past few weeks, there have been reports of variant strains of SARS-CoV-2 emerging in different parts of the world. B.1.1.7 was first identified in the UK from a sample obtained in October 2020, B.1.351 was identified in October 2020 in South Africa, and P.1 was identified in Brazil in December 2020 (https://www.cogconsortium.uk/wp-content/uploads/2021/01/Report-2_COG-UK_SARS-CoV-2-Mutations.pdf). These variant strains have picked up multiple changes (deletions and substitutions) in the spike protein, 9 in B.1.1.7, 10 in B.1.351, and 12 in P.1, compared with the Wuhan sequence. Of greatest concern are mutations in the receptor-binding domain (RBD) of the spike protein. The RBD is contained in the S1 subunit of spike and is responsible for interacting with the SARS-CoV-2 cellular receptor angiotensin-converting enzyme-2 (ACE2) (Hoffmann et al., 2020). The ACE2 interaction surface of the RBD is a relatively small 25-amino-acid patch at the tip of the RBD (Shang et al., 2020), and because of its crucial role in viral attachment, it is also the site for binding of many potent neutralizing antibodies (Cerutti et al., 2021). Blocking RBD-ACE2 interaction is thought to play a major role in natural and vaccine-induced protection from SARS-CoV-2 infection. A number of such monoclonal antibodies (mAbs) have been combined into cocktails that are in advanced trials for treatment and prophylaxis of SARS-CoV-2 (Yang et al., 2020).

The ACE2-binding surface is to some extent the Achilles heel of the virus as it can be blocked by some neutralizing antibodies; however, since it is so small, it also threatens immune escape, as small changes can throw off neutralizing antibodies, thereby reducing the ability of natural or vaccine-acquired immunity to contain viral replication. Selective pressure for changes in the ACE2 interaction surface can thus have two entirely separate drivers. First, as SARS-CoV-2 has recently crossed a zoonotic barrier, it may be expected that evolution of the ACE2 interaction surface may occur to increase affinity to ACE2 and thereby increase viral transmissibility. And second, conversely, changes to the ACE2 interaction surface may also reduce the protection afforded by previous infection or vaccination, potentially leading to escape from pre-existing immunity induced by natural infection or vaccines.

All three recently identified variant SARS-CoV-2 strains have acquired mutations in the ACE2 interaction surface of the RBD: N501Y in B.1.1.7; K417N, E484K, and N501Y in B.1.351; and K417T, E484K, and N501Y in P.1. All three variants may lead to increased transmissibility, with good evidence for this with B.1.1.7 in the UK. These have rapidly expanded to become the dominant strains in the regions where they were first identified and global spread, particularly for B.1.1.7 and B.1.351, is causing considerable concern.

Here, we examine neutralization of a B.1.351 viral isolate and compare this to neutralization of Victoria (SARS-CoV-2/human/AUS/VIC01/2020), an early Wuhan-related isolate. Neutralization assays are performed on a large panel of mAbs (Dejnirattisai et al., 2021), convalescent sera from early in the pandemic, sera from patients suffering from B.1.1.7, and finally sera from recipients of the Oxford-AstraZenca and Pfizer-BioNTech vaccines. There is evidence of widespread escape from mAbs, for which we provide a structural and biophysical description. Neutralization of B.1.351 by sera from naturally infected or vaccinated individuals is significantly reduced, leading in some cases to a complete inability to neutralize B.1.351 virus.

## Results

### Mutational changes in B.1.351

**Figure 1:**
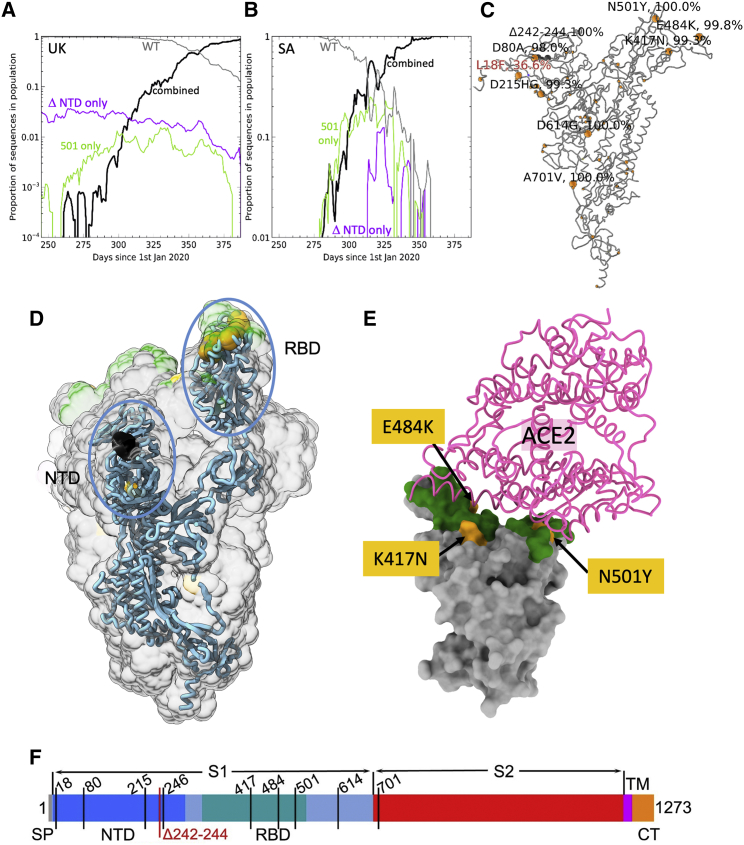
Evolution of B.1.351 variant (A and B) Sliding 7-day window depicting proportion of sequences with wild-type (gray), 501Y mutation only (green), NTD deletion only (purple), and double-mutation variant (black) for (A) sequences selected containing UK, NTD deletion 69–70 and (B) South Africa, NTD deletion 241–243. (C) Structure plot showing distribution of mutations of South African variant sequences as defined by N501Y and deletion 241–243; point mutations are marked in yellow and the deletions in dark gray. Structure plots use spike protein structure (original frame from PDB: 6ZWV) where modeled, and models were extended in Coot for missing loops. (D) Positions of major changes in the spike protein are highlighted in the NTD and RBD. (E) Positions of the K417N, E484K, and N501Y (yellow) mutations within the ACE2 interaction surface (dark green) of RBD. The view is chosen for clarity and is related to that shown in (C) by a 45° rotation around the axis coming out of the page (to make the RBD upright compared with C) and an almost 180° rotation around the long axis of the RBD domain. (F) A linear representation of B.1.351 spike with changes marked on. Note the strain used in this report does not have L18F and R246I mutations.

A number of isolates of B.1.351 have been described, all of which have the key mutations K417N, E484K, and N501Y in the RBD. Tegally et al. (2021) reported an isolate containing 10 changes relative to the Wuhan sequence: L18F, D80A, D215G, L242-244 deleted, R246I, K417N, E484K, N501Y, D614G, and A701V. Sequencing of the strain used in this report, from a case in the UK, shows only 8 changes and lacks L18F and R246I compared with the Tegally et al. (2021) isolate. Coronavirus genome sequences were analyzed in both the UK, acquired from the COVID-19 Genomics UK (COG-UK) database (Tatusov et al., 2000), and South Africa, acquired from the Global Initiative on Sharing Avian Influenza Data (GISAID) (https://www.gisaid.org). It appears that B.1.1.7 and B.1.351 quickly became overwhelmingly dominant in the UK and South Africa, respectively. In the evolution of both the B.1.1.7 variant in the UK and the B.1.351 variant in South Africa, a substantial population of N-terminal domain (NTD)-deletion-only mutants (Δ69–70 in B.1.1.7 and Δ242–244 in B.1.153), and N501Y-only mutants were observed in both countries preceding the rising dominance of strains harboring both deletions and 501Y (Figures 1A and 1B). Counts of both “single-mutant” variants have since waned. The characteristic mutations for B.1.351 as found in South Africa are shown in Figures 1C–1E. In addition, as of February 2, 2021, in the COG-UK database, 21 of the B.1.1.7 sequences were observed to have independently acquired the 484K (but not the 417N) mutation found in the B.1.351 variant, and 90 sequences display these mutations in the background of B.1.351 (as defined by bearing the characteristic Δ242–244 NTD deletion).

### Neutralization of B.1.351 by convalescent plasma

We collected plasma from a cohort of infected patients during the first wave of SARS-CoV-2 infection in the UK. Samples were collected from convalescent cases 4–9 weeks following infection in June 2020, before the emergence of B.1.1.7 (Dejnirattisai et al., 2021). We have also included a recent collection of plasma from patients infected with B.1.1.7 (Supasa et al., 2021).

**Figure 2:**
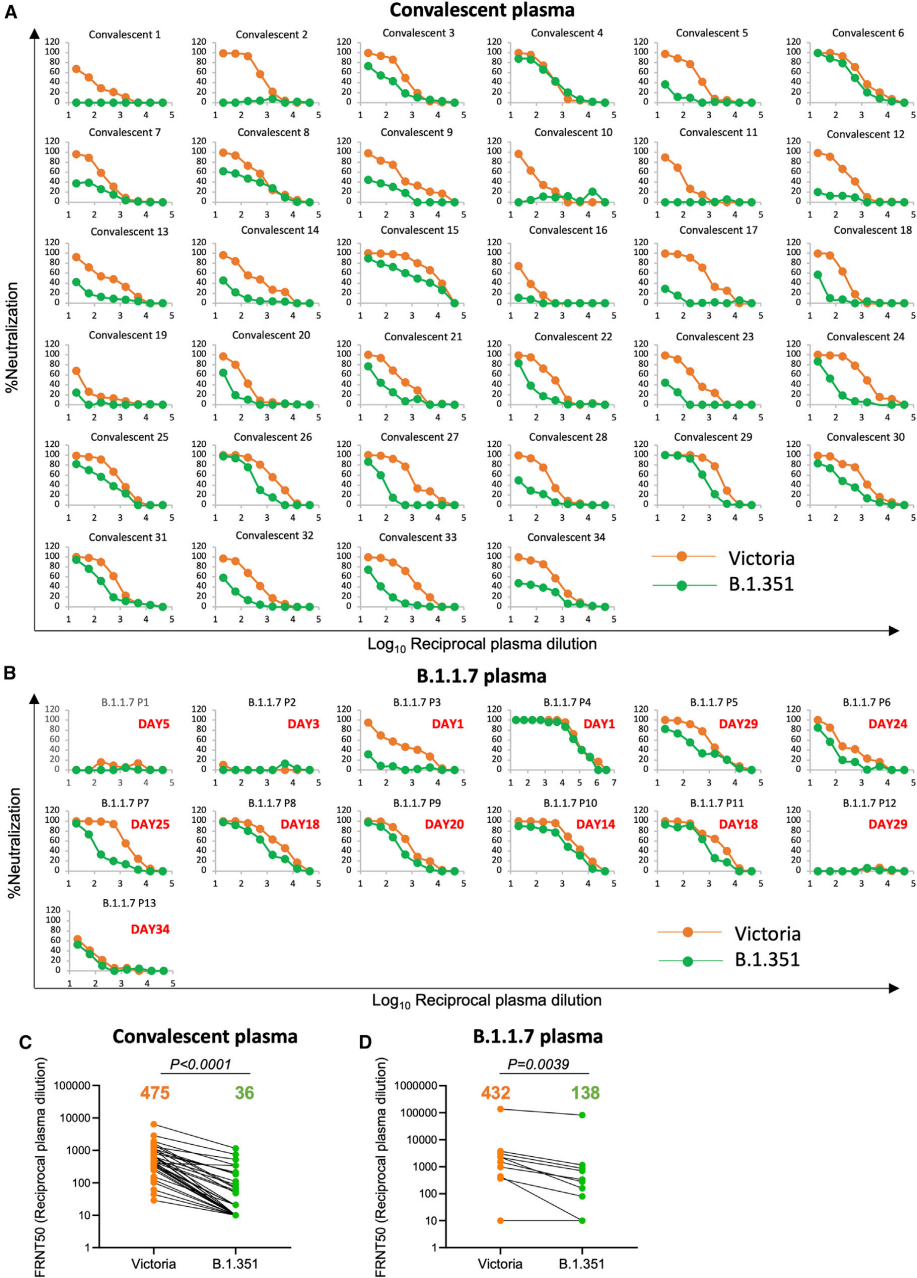
Neutralization of Victoria and B.1.351 viruses by convalescent plasma Plasma was collected in the UK before June 2020, during the first wave of SARS-CoV-2, in the early convalescent phase 4–9 weeks following admission to hospital. (A) FRNT assays comparing neutralization of Victoria (orange) and B.1.351 (green) (n = 34). (B) Neutralization assays of Victoria and B.1.351 with plasma obtained from patients suffering B.1.1.7 infection at the indicated times following infection. (C and D) Comparison of FRNT_50_ titers between B.1.351 and Victoria strains for convalescent and B.1.1.7 plasma, respectively. The Wilcoxon matched-pairs signed rank sum test was used for the analysis and two-tailed p values were calculated; geometric mean values are indicated above each column. The data underpinning the Victoria neutralization curves have been previously reported (Supasa et al., 2021). Individual FRNT_50_ values are shown in Table S1.

Neutralization titers against Victoria, an early Wuhan-related strain of SARS-CoV-2 (Caly et al., 2020; Seemann et al., 2020), were compared with B.1.351 using a focus reduction neutralization test (FRNT). For the early convalescent samples (n = 34), neutralization titers against B.1.351 were, on average, 13.3-fold reduced compared with Victoria (p < 0.0001) (Figure 2A; Table S1A). A few convalescent samples, e.g., 4, 6, and 15 retained good neutralization of B.1.351, but for most, titers were considerably reduced. Significantly, 18 of 34 samples failed to reach 50% neutralization at a plasma dilution of 1:20, with a number showing a near total reduction of neutralization activity. Overall, in the 34 convalescent plasma samples there was a 13.3-fold (geometric mean) reduction in neutralization titer between Victoria and B.1.351 (p < 0.0001) (Figure 2C).

Neutralization was also performed using plasma recently collected, at different time points, from patients suffering from B.1.1.7 (n = 13). All of these cases had S-gene knockout on diagnostic PCR (Thermo Fisher Scientific TaqPath, characteristic of B.1.1.7), and 11 had viral sequencing confirming B.1.1.7 (Figure 2B; Table S1B). Neutralization titers were low at early time points for both Victoria and B.1.351, but one case (B.1.1.7 P4), a sample taken 1 day following admission to hospital, showed a very high titer against Victoria (1:136,884) and B.1.351 (1:81,493). We speculate this may represent a reinfection with B.1.1.7. Overall, there was a 3.1-fold (geometric mean) reduction in titers between Victoria and B.1.351 in sera from patients infected with B.1.1.7 (Figure 2D).

### Neutralization of B.1.351 by vaccinee serum

**Figure 3:**
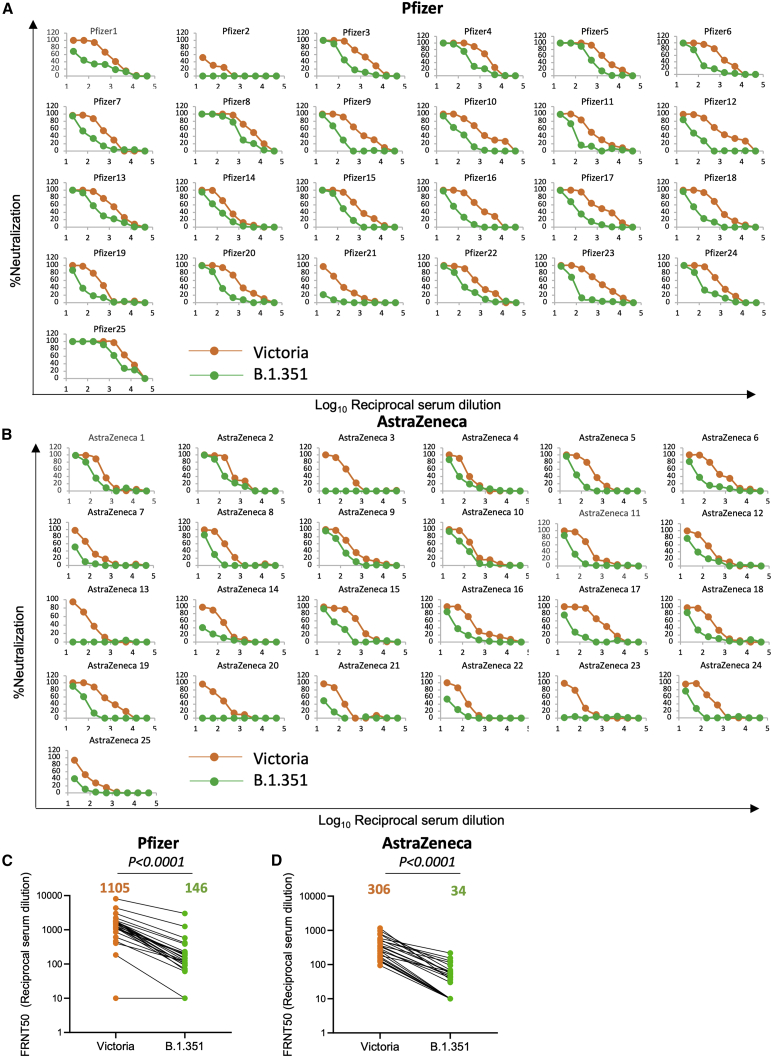
Neutralization of B.1.351 by vaccine serum (A and B) Neutralization FRNT curves for Victoria and B.1.351 strains by (A) 25 sera taken 7–17 days following the second dose of the Pfizer-BioNTech vaccine and (B) 25 sera taken 14 or 28 days following the second dose of the Oxford-AstraZeneca vaccine. (C and D) Comparison of FRNT_50_ titers between B.1.351 and Victoria strains for the Pfizer-BioNTech and Oxford-AstraZeneca vaccines, respectively. The Wilcoxon matched-pairs signed rank sum test was used for the analysis and two-tailed p values were calculated; geometric mean values are indicated above each column. The data underpinning the Victoria neutralization curves have been previously reported (Supasa et al., 2021). Individual FRNT_50_ values are shown in Table S2. See also Figure S1.

**Figure S1:**
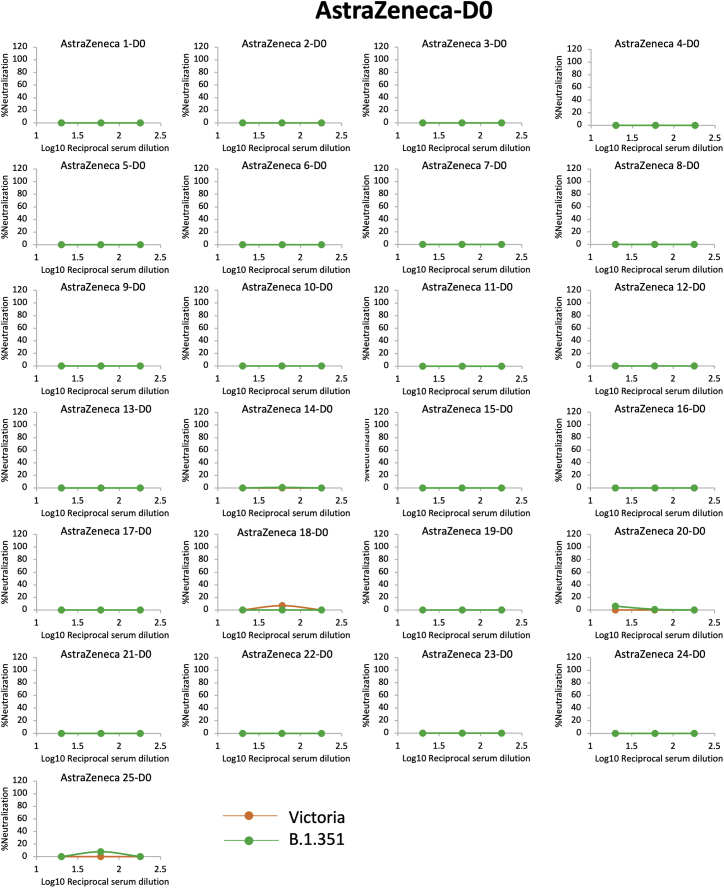
Neutralization of Victoria and B.1.351 by serum collected before vaccination, related to Figure 3 Neutralization for Victoria and B.1.351 strains by 50 sera taken at day zero before the first dose of AstraZeneca vaccine. All sera were assayed in three-fold dilutions at 1:20 and 1:60 for the final dilutions.

We next measured neutralization of Victoria and B.1.351 using vaccine serum obtained from individuals vaccinated with either the Pfizer-BioNTech vaccine BNT162b2 or the Oxford-AstraZeneca AZD1222 vaccine. For Pfizer-BioNTech, vaccinated serum was obtained from healthcare workers (n = 25) 4–17 days following the second dose of vaccine, administered 3 weeks after the first dose (Figure 3A; Table S2). For the AstraZeneca vaccine, samples (n = 25) were obtained 14 or 28 days following the second vaccine dose, with a dosing interval of 8–14 weeks (Figure 3B; Table S2). For the Pfizer-BioNTech vaccine serum, geometric mean titers for B.1.351 were 7.6-fold lower than for Victoria (p < 0.0001) (Figure 3C). For the Oxford-AstraZeneca vaccine serum, geometric mean B.1.351 titers were 9-fold lower than for Victoria (p < 0.0001) (Figure 3D; Table S2). Plasma taken pre-first dose of the Oxford-AstraZeneca vaccine showed, as expected, minimal or absent neutralization of Victoria or B.1.351 viruses (Figure S1).

The Pfizer-BioNTech vaccine serum induced 3.6-fold higher neutralization titers against the Victoria strain than the Oxford-AstraZeneca vaccine (p < 0.0001). Although the overall reduction of titers was quite similar, 7.6-fold versus 9-fold, respectively, because the AstraZeneca titers started from a lower base more of the samples failed to reach 50% FRNT titers against B.1.351 (9/25) than for the Pfizer vaccine (2/25), although one of these (Pfizer 2) also showed low neutralizing titers to the Victoria virus.

### Neutralization of B.1.351 by a large panel of mAbs

**Figure 4:**
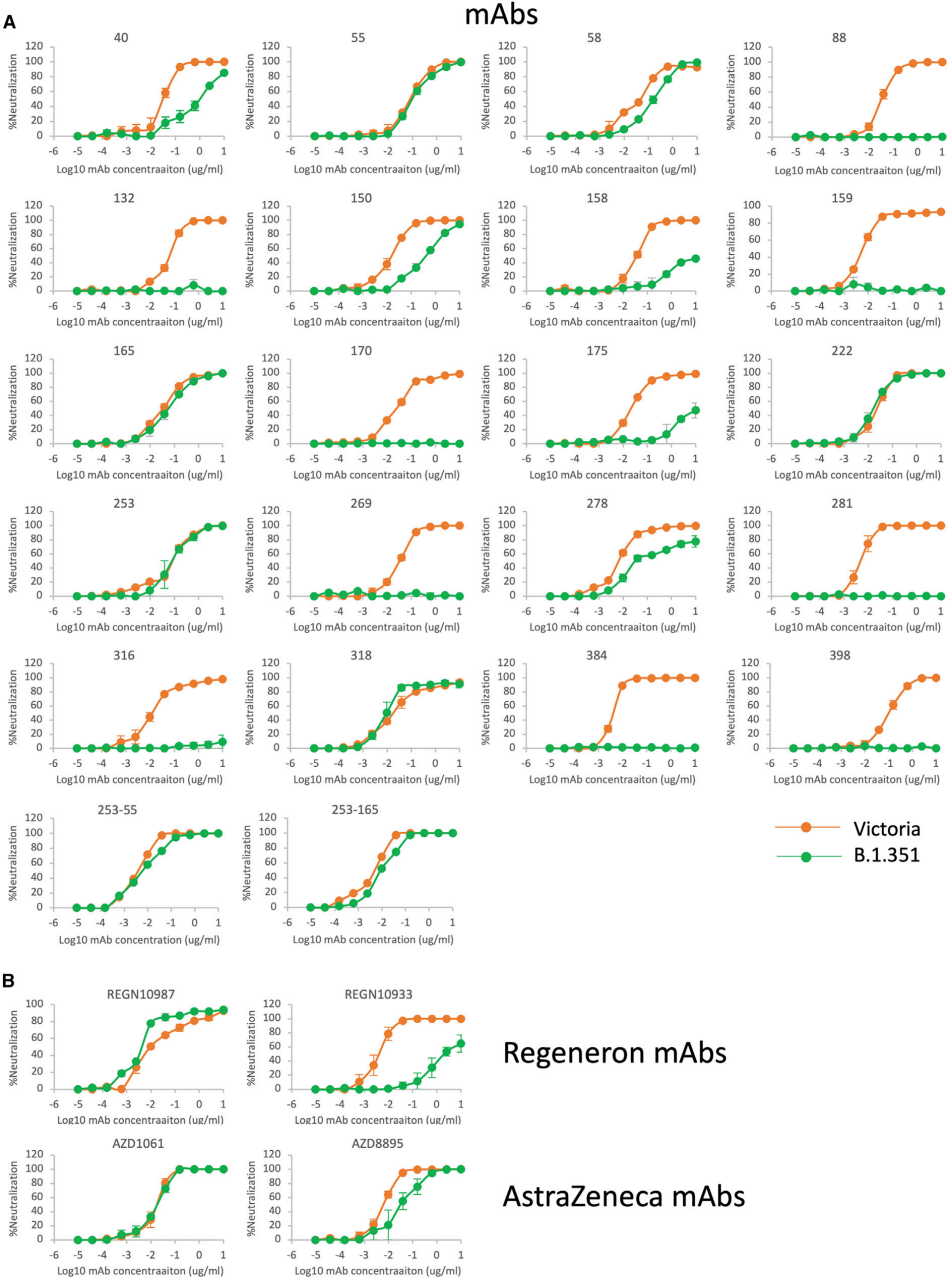
Neutralization by potent mAbs (A) Neutralization curves for Victoria and B.1.351 using 22 human monoclonal antibodies (mAbs). (B) Neutralization curves of Victoria and B.1.351 strains using mAb pairs from Regeneron and AstraZeneca. The data underpinning the Victoria neutralization curves have been previously reported (Supasa et al., 2021). Individual FRNT_50_ values are shown in Table S3.

We have produced and characterized a pool of 377 human mAbs directed to the spike protein, raised from convalescent samples obtained from patients infected during the first wave of SARS-CoV-2 in the UK before June 2020; therefore, they were not induced in response to infection with recent SARS-CoV-2 strains (Dejnirattisai et al., 2021). We selected the 20 most potent mAbs (FRNT_50_ titers < 100 ng/mL, 19 anti-RBD and 1 anti-NTD) and performed neutralization assays against Victoria and B.1.351 strains (Figure 4; Table S3A).

**Figure 5:**
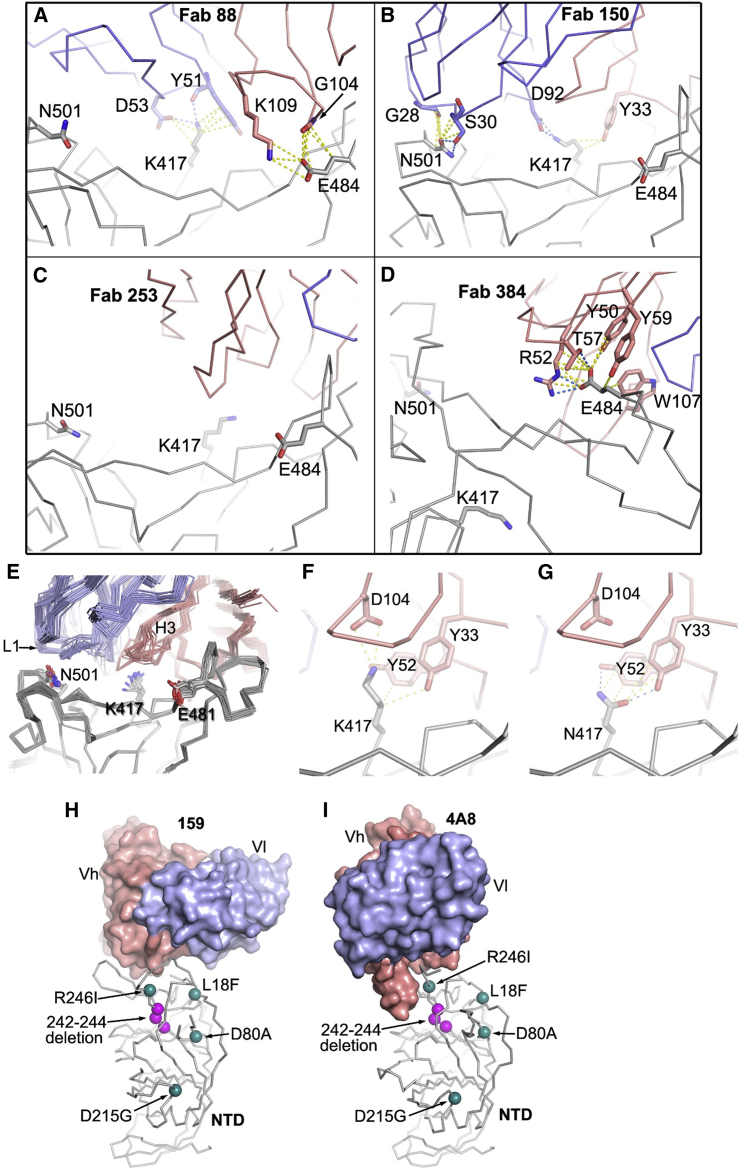
Interactions of mutation site residues with a selection of RBD-binding mAbs (A–D) Interactions of (A) Fab 88 with K417 and E484 of the RBD (PDB: 7BEL), (B) 150 with N501 and K417 (PDB: 7BEI), (C) 253 has no contact with any of the three mutation sites (PDB: 7BEN), and (D) Fab 384 with only E484 (PDB: 7BEP). (E) Structures of IGHV3-51 and IGHV3-66 Fabs by overlapping the Cα backbones of the RBD. (F) Interactions of K417 with CB6 Fab (PDB: 7C01 [Wajnberg et al., 2020]). (G) The K417N mutation is modeled in the RBD/CB6 complex. In (A) to (G), the Fab light chain, heavy chain, and RBD are in blue, salmon, and gray, respectively. Cα backbones are drawn in thinner sticks and side chains in thicker sticks. Contacts (≤4 Å) are shown as yellow dashed lines and hydrogen bonds and salt bridges as blue dashed lines. (H and I) Positions of mutations and the deletion in the spike NTD of the B.1.351 variant relative to the bound antibodies (H) 159 (PDB: 7NDC) and (I) 4A8 (PDB: 7C2L); the 242–244 deletion would be predicted to disrupt the interaction of 159 and 4A4. The heavy chain and light chain variable domains (Vh and Vl) of the Fabs are shown as salmon and blue surfaces, respectively, and the NTD as gray sticks. The mutation sites are drawn as green spheres and deletions as magenta spheres.

The effects on mAb neutralization were severe, 14 of 20 antibodies had >10-fold fall in neutralization titers, with most of these showing a complete knockout of activity. This is in line with the key roles of K417, E484, and N501, in particular E484, in antibody recognition of the ACE2 interaction surface of the RBD described below and in Figures 5A–5G.

Interestingly, the single potent NTD-binding antibody included in these analyses, mAb 159, also showed a complete knockout of activity against B.1.351, which includes deletion of amino acids 242–244 in the NTD, part of the epitope for mAb 159. As can be seen from Figures 5H and 5I, the RBD loop 246–253 interacts with the heavy chain (HC) of mAb 159 and also that of 4A8, another potent neutralizing NTD binder with a structure reported (Chi et al., 2020). The 242–244 deletion will undoubtedly alter the presentation of this loop, compromising binding to these mAbs. Binding at this so-called “supersite” has been reported as of potential therapeutic relevance (McCallum et al., 2021). The B.1.1.7, B.1.351, and P.1 lineages have all converged with either deletions or systematic changes in the NTD. Although P.1 does not harbor NTD deletions, the changes L18F, T20N, and P26S (Faria et al., 2021) would be expected to impact markedly on binding at the NTD epitope. Since these convergent features may have arisen prior to strong selective pressure from antibody responses, it seems likely there is an underlying biological driver still to be discovered, like the increased receptor binding and potential increased transmissibility imparted by the RBD mutations, which may cause this epitope to be extremely susceptible to mutation and escape from antibody binding.

### Neutralization of B.1.351 by mAbs in late-stage clinical trials

A number of mAbs are in late-stage clinical trials as therapy or prophylaxis against SARS-CoV-2 (Ku et al., 2021; Baum et al., 2020). Regeneron and AstraZeneca use cocktails of 2 mAbs to give resistance to mutational escape of viruses (Kemp et al., 2021). We performed neutralization assays with the Regeneron pair REGN10933 and REGN10987 and the AstraZeneca pair AZD106 and AZD8895 (Figure 4B; Table S3B). The neutralization of REGN10987 was unaffected by B.1.351, while REGN10933 was severely impaired (773-fold) (Figure 4B; Table S2). Neutralization by the AZ pair of antibodies was little affected on B.1.351 compared with Victoria.

### Understanding the abrogation of neutralization: ACE2 binding to B.1.351 RBD

**Figure 6:**
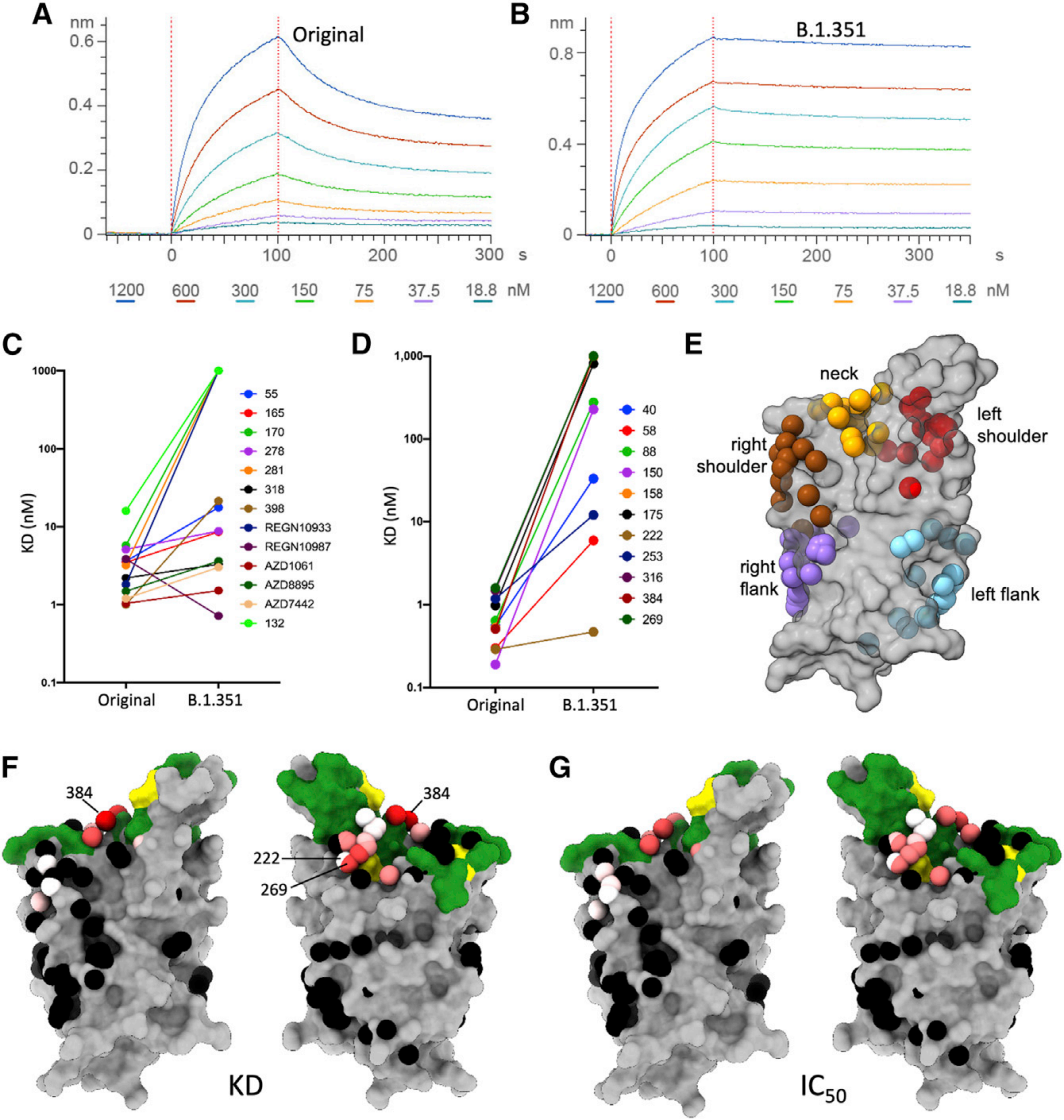
Antibody RBD interaction and structural modeling (A and B) BLI plots showing a titration series of binding to ACE2 (see STAR Methods) for (A) Wuhan RBD and (B) K417N, E484K, and N501Y B.1.351 RBD. Note the much slower off-rate for B.1.351. (C and D) K_D_ of RBD/mAb interaction measured by BLI for WT Wuhan RBD (left dots) and K417N, E484K, and N501Y B.1.351 RBD (right dots). (E) Epitopes as defined by the clustering of mAbs on the RBD (gray). (F) BLI data mapped onto the RBD using the method described in (Dejnirattisai et al., 2021). Front and back views of the RBD are depicted with the spheres representing the antibody binding sites colored according to the ratio (K_D_B.1.351/K_D_Wuhan). For white, the ratio is 1; for red, it is <0.1 (i.e., at least 10-fold reduction). Black dots refer to mapped antibodies not included in this analysis; dark green to RBD ACE2-binding surface; and yellow to mutated K417N, E484K, and N501Y. (G) As for the left pair, but colored according to the ratio of neutralization titers (half-maximal inhibitory concentration [IC_50_]B.1.351/[IC_50_]Victoria). For white, the ratio is 1; for red, it is <0.01 (i.e., at least 100-fold reduction). Note the strong concordance between the two effects, with 269 being the most strongly affected. The nearby pink antibodies are mainly the IGHV3-53 and IGHV3-66 antibodies.

The triple mutation K417N, E484R, and N501Y is characteristic of the B.1.351 RBD. These residues are situated within the ACE2 footprint (Figure 1E), and *in vitro* evolution to optimize the affinity for ACE2 has suggested that they confer higher affinity for the receptor (Starr et al., 2020; Zahradník et al., 2021). To investigate this effect, we measured the kinetics of binding of soluble ACE2 to recombinant RBD by biolayer interferometry (BLI) (Figures 6A and 6B). As expected, the affinity for B.1.351 RBD is high; in fact, it is 19-fold higher than for the Victoria RBD and 2.7-fold higher than for B.1.1.7 ((Dejnirattisai et al., 2021); Supasa et al., 2021). The K_D_ is 4.0 nM, K_on_ is 4.78E4/Ms, and K_off_ is 1.93E−4/s; thus, the off-rate is approximately 1.5 h. This will further exacerbate the decline in potency observed in neutralization assays, since antibodies of lower affinity will struggle to compete with ACE2 unless they have a very slow off-rate or show an avidity effect to block attachment. Thus, while all of our set of potent RBD binders have an affinity higher than that between ACE2 and Victoria or B.1.1.7 RBD (K_D_s of 75.1 and 10.7 nM, respectively), five of the 19 have lower or equal affinity than for ACE2 and B.1.351 RBD. A small further increase in affinity (e.g., 2-fold) would beat almost all the antibodies ((Dejnirattisai et al., 2021); Supasa et al., 2021).

### The influence of RBD mutations on mAb affinity

To understand the order of magnitude of the abrogation in neutralization of more than two-thirds of the 19 potent mAbs that bind the RBD, we measured the K_D_ for binding to recombinant RBD by BLI (Figures 6C and 6D; Table S3). The results are stark: whereas for the antigen binding fragments (Fabs) tested against Victoria, 17 had K_D_s below 4 nM (the affinity of ACE2 for B.1.351) against B.1.351, this reduced to 4 (or 2 if the engineered light-chain [LC] versions of 253 are removed [(Dejnirattisai et al., 2021)]), with 7 Fabs failing to achieve near-micromolar affinity. These results broadly follow the neutralization results (compare panels C and D of Figure 6; see Table S3), suggesting that the observed pattern of effects on neutralization is largely due to the amino acid substitutions in the RBD, that is, K417N, E484K, and N501Y.

### The structural basis for loss of mAb binding

We attempt to understand the basis of these effects in the context of an anatomical description of the RBD. In terms of a human torso, we have defined four almost contiguous structural epitopes: left shoulder, neck, right shoulder, and right flank, with a separate left flank epitope (Dejnirattisai et al., 2021) (Figure 6E). In this context, the ACE2-binding site extends across the neck and both shoulders. N501Y is on the right shoulder, K417N at the back of the neck, and E484R on the left shoulder. Although the three mutations are nominally in different epitopes, the overlapping nature of these epitopes means that the residues are sufficiently close so that more than one might directly affect the binding of any one antibody. In addition, there may be allosteric effects (the structural equivalent of epistasis in genetics) whereby effects may extend over some distance. Despite these caveats, the majority of the effects observed are directly explicable by reference to prior structural knowledge.

### The effect of N501Y and K417N on mAb binding

Many of the reported Fab/SARS-CoV-2 RBD complexes are for antibodies that use the public immunoglobulin HC variable (IGHV) region IGHV3-53 ((Dejnirattisai et al., 2021); Yuan et al., 2020), and these are well represented in our set by five antibodies that are potent against the Victoria virus. Four of these, 150, 158, 175, and 269, have their neutralization and binding abilities severely compromised or abolished, while 222 is an exception, since its binding is unaffected by the B.1.351 variant (Figures 6F and 6G). The family of IGHV3-53 antibodies bind at the same epitope at the back of the neck of the RBD, with very similar approach orientations also shared by the IGHV3-66 Fabs. The majority of these make direct contacts to K417 and N501, but none of them contact E484. The rather short HC complementarity-determining regions-3 (CDR3s) of these Fabs are usually positioned directly above K417 (Dejnirattisai et al., 2021), making hydrogen bonds or salt bridges as well as hydrophobic interactions, while N501 interacts with the LC CDR1 loop (Figure 5) (Supasa et al., 2021). However, mAb 150 is a little different, forming both a salt bridge between K417 and the LC CDR3 D92 and a hydrogen bond between N501 and S30 in the LC CDR1 (Figure 5B), whereas 158 is more typical, making a hydrogen bond from the carbonyl oxygen of G100 of the HC CDR3 and K417 and hydrophobic contacts from S30 of the LC CDR1 to N501. We would therefore expect that the combined effects of the K417N and N501Y mutations would severely compromise the binding of most IGHV3-53 and IGHV3-66 class mAbs. However, one member of this class, 222, is unaffected by either the B.1.1.7 (Supasa et al., 2021) or the B.1.351 variant. Unfortunately, to date we have been unable to obtain a structure of the 222 Fab with RBD or spike. However, we have previously noted that p2c-2f11 Fab (PDB: 7CDI [Wajnberg et al., 2020]) whose LC is most similar in sequence, and has the same CDR-L1, L2, and L3 lengths, to mAb 222 does not make any contact with N501 (Supasa et al., 2021).

By closely examining the structures of the IGHV3-53 and IGHV3-66 Fab and RBD complexes, we found that Fab CB6 (PDB: 7C01 [Shi et al., 2020]) has the same CDR-H1-3 lengths and only makes hydrophobic contacts to K417 from Y33, Y52, and D104 of the HC. Changing the K to N at 417 in this complex structure and selecting one of the favorable side-chain rotamers shows that N417 could make hydrogen bonds to both Y33 and Y52, compensating for the loss of contact to D104 (Figures 5F and 5G). In 222, Y33 and Y52 are conserved and D104 is replaced by an N. We speculate that the interaction of 222 with K417 might be similar to CB6, explaining its resistance to the B.1.351 variant.

### The effects of the E484K mutation

Fab 88 binds RBD at the back of the left shoulder, residues G104 and K108 of the HC CDR3 contact E484, and the LC CDR2 makes extensive hydrophobic interactions and a main-chain hydrogen bond from Y51 and a salt bridge from D53 to K417 (Figure 5A). The change of charge at E484 from negative to positive and shortening of the residue 417 side chain from K to N would be expected to abolish all these interactions, explaining the several hundred-fold loss in K_D_. mAb 384 is one of the most potent neutralizing mAbs we have found against the Victoria virus. This mAb approaches the binding site from the front of the left shoulder, burying 82% of the solvent-accessible area of E484 by hydrogen bonding with Y50, T57, and Y59 as well as making a salt bridge with R52 of the LC CDR2 (Figure 5D), thereby explaining the catastrophic impact of the E484K mutation on binding (Table S3).

### Antibodies resistant to K417N, E484K, and N501Y

mAb 222 was not the only antibody to show resilience to B.1.351. The FRNT_50_ titers for mAbs 55, 165, 253, and 318 were also relatively equal between Victoria and B.1.351, indicating that their epitopes are not perturbed by the K417N, E484K, and N501Y mutations. Antibodies 55, 165, and 253 are related to each other, and we have previously shown that combining the LCs of 55 or 165 with the HC of 253 leads to a >1 log increase in neutralization titers (Dejnirattisai et al., 2021). The chimeras 253H/55L and 253H/165L can both neutralize B.1.351, with FRNT_50_ titers of 9 and 13 ng/mL, respectively. Structures of 253 and these chimera Fabs with either RBD or spike show that they bind almost identically to the same epitope and do not contact any of the three mutation site residues, correlating well with the neutralization and BLI binding data (Figure 5C).

## Discussion

Coronaviruses are positive-stranded RNA viruses, and although the RNA polymerase possesses limited proofreading capacity, they are intrinsically prone to mutational change. The evolution of SARS-CoV-2 from a likely single-point zoonotic introduction in Wuhan in November or December 2019 has been widely anticipated and indeed led to the establishment of viral sequence surveillance such as COG-UK in the UK. Similar surveillance efforts have been started in a number of countries, but globally coverage is insufficient, with large at-risk populations with little or no capacity.

The recent emergence of three strains of SARS-CoV-2, B.1.1.7, B1.351, and P.1, which may impart increased transmissibility, has occurred independently in the UK, South Africa, and Brazil, where they have rapidly become dominant strains and are now spreading globally. While the sequences are markedly different, each containing 9–12 changes, there are two common themes. The first theme involves the ACE2 interaction surface of the RBD: all share the N501Y mutation, while B.1.351 and P.1 share E484K and N501Y and both B.1.351 and P.1 have changes at 417, 417T in P.1 and 417N in B.1.351. The second theme is deletions in the NTD: 69–70 and 144 in B.1.1.7 and 242–244 in B.1.351, both of which will disrupt the binding sites of neutralizing anti-NTD antibodies, as shown here by the failure of neutralization of anti-NTD mAb 159. Although P.1 does not possess NTD deletions, it is studded with point mutations in this region that may confer similar functional properties. Despite the changes in B.1.351, residual neutralizing capacity is present in many convalescent and vaccine sera, with some individuals showing minimal reduction of titers relative to the Victoria strain.

Although the majority of potent mAbs suffered substantial reduction or knockout of activity, a number were able to potently neutralize B.1.351, including 222, 318, 253/55, and 353/165, the AstraZeneca pair AZD1061 and AZD889, and Regeneron REGN10987, which does not contact any of the mutation sites, whereas REGN10933 contacts both 417 and 484 and binding is abrogated. By analysis of known structures of Fab/RBD complexes, we are able to rationalize the effects on all potent binders to the Victoria virus in terms of interactions with the three mutated residues in the RBD.

How much further mutation in the RBD these antibodies will be able to withstand is not known, but the use of cocktails of antibodies to hedge against viral variants occurring either during a single infection, or at a population level, appears to be a sound strategy. However, it must be recognized that the use of mAb therapy or prophylaxis, particularly for extended periods in chronically infected immunocompromised individuals, is likely to drive the emergence of resistance mutations.

The widespread emergence of variant strains, particularly containing the E484K mutation, may make it prudent to develop mAbs to target the 484K change. It may also be possible to re-engineer existing candidate therapeutic antibodies; an example of how subtle changes can confer resilience is shown by mAb 222, which possesses the IGHV3-53 V-region. While all other IGHV3-53 antibodies are severely compromised in binding B.1.351, a slight change in the length of the HC CDR3 and a suitable choice of LC enable mAb 222 to maintain potency against B.1.1.7 and B.1.351.

*In vitro* evolution experiments have recently been reported (Starr et al., 2020) in which live virus has been induced to evolve in the face of immune pressure from either mAbs or polyclonal serum. Interestingly, repeated use of plasma therapy in an immunocompromised individual led to the transient emergence of the N501Y mutation as well as the 69–70 deletion in the NTD, which is characteristic of B.1.1.7 (Kemp et al., 2021). Furthermore, serial passage of virus in sub-neutralizing concentrations of immune plasma led to the emergence of the deletion of F140 and the creation of a new N-linked glycosylation sequon in the NTD together with the E484K RBD mutation (Andreano et al., 2020).

Alternatively, yeast display of libraries of RBD mutants has been used to select variants for escape from binding to immune serum or alternatively for increased affinity of binding to the ACE2 receptor (Zahradník et al., 2021). Of great interest is that all of these approaches have led to the identification of a common set of mutations found in the variant viruses that are now circulating. Principal among these are N501Y found in all B.1.1.7, B.1.351, and P.1 lineages and E484K found in B.1.351 and P.1. These mutations increase the affinity of the RBD for ACE2 2.7-fold for B.1.1.7 (Supasa et al., 2021) and 19-fold for B.1.351, which is compatible with the observation that viruses carrying the E484K and N501Y mutations likely have increased transmissibility.

Here, we demonstrate that the B.1.351 CoV-2 strain is much more difficult to neutralize than parental strains; 14 of 20 of a panel of mAbs are seriously compromised or neutralization is completely knocked out. On convalescent serum, the neutralization titers are reduced 13.3-fold for B.1.351 compared with the Victoria strain, with 14 of 34 failing to reach an NT_50_ at a 1:20 dilution and a number showing almost complete knockdown of activity. It remains to be determined whether this reflects a focusing of the immune response in these individuals, as has been seen, for instance, for the picornavirus enterovirus-71 (Huang et al., 2020). Neutralization titers for the Oxford-AstraZeneca and Pfizer vaccines were similarly reduced with B.1.351 by 9-fold and 7.6-fold, respectively. For the Oxford-AstraZeneca vaccine compared with the Pfizer vaccine, more sera failed to reach FRNT_50_ at 1:20 dilution, and since the reduction in FRNT_50_ titers between the two vaccines was quite similar, this effect was due to the 3.6-fold lower starting titers for the Oxford-AstraZenca vaccine versus the Pfizer-BioNTech vaccine. However, both the Oxford-AstraZeneca and Pfizer vaccines give substantial initial efficacy after a single dose of vaccine against parental strains (∼76 and 89%, respectively) (https://assets.publishing.service.gov.uk/government/uploads/system/uploads/attachment_data/file/961287/Greenbook_chapter_14a_v7_12Feb2021.pdf; Baden et al., 2021; Polack et al., 2020, (Voysey et al., 2021b)), implying neutralizing antibody titers required for this level of protection are modest.

Very recent data suggest that the Novavax vaccine, which achieved 95.6% efficacy against previous SARS-CoV-2 strains and 85.6% against B.1.1.7 in the UK, had reduced efficacy of 60% in South Africa, where 92.6% of infections are estimated to have been B.1.351 (https://www.novavax.com/sites/default/files/2021-02/20210202-NYAS-Novavax-Final.pdf). Furthermore, data from the Novavax trial in South Africa indicate that approximately one-third of the study participants were seropositive at enrollment; however, in the placebo arm of the study, there was no difference in the rate of infection in seronegative versus seropositive volunteers (3.9 versus 3.9%), implying a lack of protection of previous SARS-CoV-2 exposure to infection with B.1.351. The Janssen single-dose COVID-19 vaccine showed 72% efficacy at preventing moderate and severe disease, which was reduced to 57% in South Africa. Finally, a recent report from South Africa on a small sample size suggests substantial loss of efficacy for the Oxford-AstraZeneca vaccine against B.1.351 infection (10.6% efficacy against mild-to-moderate disease [Madhi et al., 2021]). There are no reports yet of the efficacy of the Pfizer-BioNTech vaccine against B.1.351; however, the neutralization titers reported here suggest that a degree of efficacy will be retained. Overall, these results suggest that previous infection or vaccination with ancestral strains of SARS-CoV-2 may not provide adequate protection against B.1.351.

What is driving the evolution of B.1.351 is difficult to disentangle. On the one hand, we show here an ∼20-fold increase in affinity for ACE2 compared with Wuhan RBD, which may influence transmissibility. On the other hand, the substantial antibody immune escape by B.1.351 is likely playing a role in countries such as South Africa, where the rates of previous infection are relatively high (estimate >30%). The trade-off of increased ACE2 affinity and transmissibility against immune escape is likely complex; as population immunity increases due to vaccination and natural infection, the evolutionary pressure for viral variants to be selected ratchets up. The ability to generate ultra-high-affinity RBD variants for ACE2 in the sub-picomolar range by *in vitro* evolution (Zahradník et al., 2021), a higher affinity than almost all mAbs described to date, is a cause for concern. Whether such viruses with extreme ACE2/RBD affinity are viable is clearly unknown, and extreme caution should be exercised as to whether this scenario should ever be tested using live viruses.

In summary, the recent emergence of multiple variant strains of SARS-CoV-2 has disrupted confidence around whether the current generation of vaccines will provide long-term protection against infection. The possibility of escape from natural and vaccine-induced immunity has prompted a rush to understand the consequences of these changes and spurred a push to develop new vaccine constructs tailored to the variants, particularly incorporating the E484K mutation. How previously infected or vaccinated individuals respond to these new variant vaccines will be the subject of intense study over the coming months, as there is a general reckoning that the current problem is not over. However, even if antibody responses to the new variants are not able to prevent infection, they may moderate severity. In addition, T cell responses to spike may not be disrupted by the mutational changes and be able to limit spread to the lower respiratory tract and prevent severe disease.

Intensive surveillance systems need to be implemented to monitor for the emergence of new variants and, in particular, to be targeted at searching for breakthrough infections in vaccinees. Work on second and even third generation vaccines to target variant viruses and more broadly to develop immunogens to targets less reliant on the ACE2-RBD interaction surface are deserving of further study.

### Limitations of the study

The vaccine and convalescent samples used in the neutralization studies in this report were taken early and titers may rise further; conversely, it is also likely that titers will wane with time and that protection from B.1.351 afforded by antibody responses to early SARS-CoV-2 strains may reduce. It will also be important to know how serum from individuals infected with B.1.351 is able to neutralize early Wuhan-related strains as well as the recently reported variants B.1.1.7 and P.1. Furthermore, since E484K appears to be such an important mutation with respect to antibody binding and neutralization, future studies may seek to define mAbs from individuals infected with E484K viruses to provide protection from these virus strains that are being pressured to emerge, we believe mainly through increased fitness imparted by the higher affinity of RBD for ACE2. Finally, it will be important to determine whether vaccination or natural infection with early strains of SARS-CoV-2 still affords protection from severe disease and hospitalization, the most important metrics of vaccine success.

## STAR★Methods

### Key resources table

**Table:** 

REAGENT or RESOURCE	SOURCE	IDENTIFIER
**Antibodies**		

Fab	(Dejnirattisai et al., 2021)	N/A
IgG	(Dejnirattisai et al., 2021)	N/A
Human anti-NP (mAb 206)	(Dejnirattisai et al., 2021)	N/A
Regeneron mAbs	AstraZeneca	Cat#REGN10933, and REGN10987
AstraZeneca mAbs	AstraZeneca	Cat#AZD1061, AZD8895
Anti-Human IgG (Fc specific)-Peroxidase	Sigma	Cat#A0170 RRID: AB_257868

**Bacterial and virus strains**		

SARS-CoV-2 (Australia/VIC01/2020)	Caly et al., 2020	N/A
SARS-CoV-2/B.1.1.7	Public Health England	N/A
DH5α bacteria	*In Vitro*gen	Cat#18263012

**Biological samples**		

Serum from Pfizer-vaccinated individuals	University of Oxford	N/A
Serum from AstraZeneca-Oxford-vaccinated individuals	University of Oxford	N/A
Plasma from SARS-CoV-2 patients	John Radcliffe Hospital in Oxford UK	N/A

**Chemicals, peptides, and recombinant proteins**		

His-tagged SARS-CoV-2 RBD	This paper	N/A
His-tagged SARS-CoV-2 RBD K417N, E484K, N501Y	This paper	N/A
His-tagged human ACE2	This paper	N/A
Human ACE2-hIgG1Fc	This paper	N/A
Phosphate buffered saline tablets	Sigma-Aldrich	Cat#P4417
Dulbecco’s Modified Eagle Medium, high glucose	Sigma-Aldrich	Cat#D5796
Dulbecco’s Modified Eagle Medium, low glucose	Sigma-Aldrich	Cat#D6046
FreeStyle 293 Expression Medium	GIBCO	Cat#12338018
L-Glutamine–Penicillin–Streptomycin solution	Sigma-Aldrich	Cat#G1146
Fetal Bovine Serum	GIBCO	Cat#12676029
Polyethylenimine, branched	Sigma-Aldrich	Cat#408727
Carboxymethyl cellulose	Sigma	Cat#C4888
Strep-Tactin®XT	IBA Lifesciences	Cat#2-1206-025
HEPES	Melford	Cat#34587-39108
Sodium Chloride	Honeywell	Cat#SZBF3340H
LB broth	Fisher Scientific UK	Cat#51577-51656
Mem Neaa (100X)	GIBCO	Cat#2203945
Trypsin-EDTA	GIBCO	Cat#2259288
L-Glutamine 200 mM (100X)	GIBCO	Cat#2036885
SYPROorange (5000X in DMSO)	Thermo	Cat#S6651
Isopropyl β-d-1-thiogalactopyranoside	Meridian Bioscience	Cat#BIO-37036
Kanamycin	Melford	Cat#K22000
Lysozyme	Sigma-Aldrich	Cat#L6876
Tris-base	Melford	Cat#T60040
Imidazole	Sigma-Aldrich	Cat#56750
Triton X-100	Sigma-Aldrich	Cat#8787
Turbonuclease	Sigma-Aldrich	Cat#T4330
RNase A	QIAGEN	Cat#158922
NaCl	Sigma-Aldrich	Cat#S9888
MgSO4	Sigma-Aldrich	Cat#746452
Na2HPO4	Melford	Cat#S23100
NaH2PO4	Melford	Cat#S23185

**Experimental models: cell lines**		

HEK293S GnTI- cells	ATCC	Cat#CRL-3022 RRID: CVCL_A785
HEK293 cells	ATCC	Cat#CRL-3216 RRID: CVCL_0063
Expi293F Cells	GIBCO,	Cat#A14527
Hamster: ExpiCHO cells	Thermo Fisher	Cat#A29133
Vero cells	ATCC	Cat#CCL-81 RRID: CVCL_0059

**Recombinant DNA**		

Vector: pHLsec	(Aricescu et al., 2006)	N/A
Vector: pNEO	(Aricescu et al., 2006)	N/A
Vector: pOPING-ET	(Nettleship et al., 2008)	N/A
human ACE2 cDNA	Sourcebiosciences	Cat#5297380
Vector: human IgG1 heavy chain	German Cancer Research Center, Heidelberg, Germany (H. Wardemann	N/A
Vector: human lambda light chain	German Cancer Research Center, Heidelberg, Germany (H. Wardemann	N/A
Vector: human kappa light chain	German Cancer Research Center, Heidelberg, Germany (H. Wardemann	N/A
Vector: Human Fab	Univeristy of Oxford	N/A
Vector: Human scFv	University of Oxford, NDM (G. Screaton)	N/A

**Software and algorithms**		

PyMOL	Schrodinger	https://pymol.org/2/; RRID: SCR_000305
Data Acquisition Software 11.1.0.11	Fortebio	https://www.sartorius.com/en/products/protein-analysis/octet-systems-software
Data Analysis Software HT 11.1.0.25	Fortebio	https://www.sartorius.com/en/products/protein-analysis/octet-systems-software
Prism 8.0	GraphPad	https://www.graphpad.com/scientific-software/prism/ RRID: SCR_002798
IBM SPSS Software 26	IBM	https://www.ibm.com/us-en/?ar=1 RRID: SCR_019096
mabscape	This paper	https://github.com/helenginn/mabscape https://snapcraft.io/mabscape

**Other**		

TALON® Superflow Metal Affinity Resin	Clontech	Cat#635668
HiLoad® 16/600 Superdex® 200 pg	Cytiva	Cat#28-9893-35
Superdex 200 increase 10/300 GL column	Cytiva	Cat#28990944
HisTrap HP 5-ml column	Cytiva	Cat#17524802
HiTrap Heparin HT 5-ml column	Cytiva	Cat#17040703
Amine Reactive Second-Generation (AR2G) Biosensors	Fortebio	Cat#18-5092
Octet RED96e	Fortebio	https://www.sartorius.com/en/products/protein-analysis/octet-label-free-detection-systems
Buffer exchange system “QuixStand”	GE Healthcare	Cat#56-4107-78
Sonics vibra-cell vcx500 sonicator	VWR	Cat#432-0137

### Resource availability

#### Lead contact

Resources, reagents and further information requirement should be forwarded to and will be responded by the Lead Contact, David I Stuart (dave@strubi.ox.ac.uk).

#### Materials availability

Reagents generated in this study are available from the Lead Contact with a completed Materials Transfer Agreement.

#### Data and code availability

Mabscape is available from https://github.com/helenginn/mabscape, https://snapcraft.io/mabscape. The data that support the findings of this study are available from the corresponding authors on request.

### Experimental model and subject details

#### Viral stocks

SARS-CoV-2/human/AUS/VIC01/2020 (Caly et al., 2020) and SARS-CoV-2/B.1.351, provided by Public Health England, were both grown in Vero (ATCC CCL-81) cells. Cells were infected with the SARS-CoV-2 virus using an MOI of 0.0001. Virus containing supernatant was harvested at 80% CPE and spun at 2000 rpm at 4°C before storage at −80°C. Viral titers were determined by a focus-forming assay on Vero cells. Both Victoria passage 5 and B.1.351 passage 4 stocks were sequenced to verify that they contained the expected spike protein sequence and no changes to the furin cleavage sites. The B1.351 virus used in these studies contained the following mutations: D80A, D215G, L242-244 deleted, K417N, E484K, N501Y, D614G, A701V.

#### Bacterial Strains and Cell Culture

Vero (ATCC CCL-81) cells were cultured at 37°C in Dulbecco’s Modified Eagle medium (DMEM) high glucose (Sigma-Aldrich) supplemented with 10% fetal bovine serum (FBS), 2 mM GlutaMAX (GIBCO, 35050061) and 100 U/ml of penicillin–streptomycin. Human mAbs were expressed in HEK293T cells cultured in UltraDOMA PF Protein-free Medium (Cat# 12-727F, LONZA) at 37°C with 5% CO_2_. *E.coli DH5α* bacteria were used for transformation of plasmid pNEO-RBD K417N, E484K, N501Y. A single colony was picked and cultured in LB broth with 50 μg mL^-1^ Kanamycin at 37°C at 200 rpm in a shaker overnight. HEK293T (ATCC CRL-11268) cells were cultured in DMEM high glucose (Sigma-Aldrich) supplemented with 10% FBS, 1% 100X Mem Neaa (GIBCO) and 1% 100X L-Glutamine (GIBCO) at 37°C with 5% CO_2_. To express RBD, RBD K417N, E484K, N501Y and ACE2, HEK293T cells were cultured in DMEM high glucose (Sigma) supplemented with 2% FBS, 1% 100X Mem Neaa and 1% 100X L-Glutamine at 37°C for transfection.

#### Participants

Participants were recruited through three studies: Sepsis Immunomics [Oxford REC C, reference:19/SC/0296]), ISARIC/WHO Clinical Characterization Protocol for Severe Emerging Infections [Oxford REC C, reference 13/SC/0149] and the Gastro-intestinal illness in Oxford: COVID sub study [Sheffield REC, reference: 16/YH/0247]. Diagnosis was confirmed through reporting of symptoms consistent with COVID-19 and a test positive for SARS-CoV-2 using reverse transcriptase polymerase chain reaction (RT-PCR) from an upper respiratory tract (nose/throat) swab tested in accredited laboratories. A blood sample was taken following consent at least 14 days after symptom onset. Clinical information including severity of disease (mild, severe or critical infection according to recommendations from the World Health Organization) and times between symptom onset and sampling and age of participant was captured for all individuals at the time of sampling.

#### Sera from Pfizer vaccinees

Pfizer vaccine serum was obtained 7-17 days following the second dose of vaccine which was administered 3 weeks after the first dose (participants were to the best of their knowledge seronegative at entry).

The study was approved by the Oxford Translational Gastrointestinal Unit GI Biobank Study 16/YH/0247 [research ethics committee (REC) at Yorkshire & The Humber – Sheffield]. The study was conducted according to the principles of the Declaration of Helsinki (https://www.wma.net/policies-post/wma-declaration-of-helsinki-ethical-principles-for-medical-research-involving-human-subjects/) and the International Conference on Harmonization (ICH) Good Clinical Practice (GCP) guidelines. Written informed consent was obtained for all patients enrolled in the study. Vaccinees were Health Care Workers, based at Oxford University Hospitals NHS Foundation Trust, not known to have prior infection with SARS-CoV-2. Each received two doses of COVID-19 mRNA Vaccine BNT162b2, 30 μg, administered intramuscularly after dilution as a series of two doses (0.3 mL each) 18-28 days apart. The mean age of vaccines was 43 years (range 25-63), 11 male and 14 female.

#### AstraZeneca-Oxford vaccine study procedures and sample processing

Full details of the randomized controlled trial of ChAdOx1 nCoV-19 (AZD1222), were previously published (PMID: 33220855/PMID: 32702298). These studies were registered at ISRCTN (15281137 and 89951424) and ClinicalTrials.gov (NCT04324606 and NCT04400838). Written informed consent was obtained from all participants, and the trial is being done in accordance with the principles of the Declaration of Helsinki (2008) and Good Clinical Practice. The studies were sponsored by the University of Oxford (Oxford, UK) and approval obtained from a national ethics committee (South Central Berkshire Research Ethics Committee, reference 20/SC/0145 and 20/SC/0179) and a regulatory agency in the United Kingdom (the Medicines and Healthcare Products Regulatory Agency). An independent DSMB reviewed all interim safety reports. A copy of the protocols was included in previous publications (PMID: 33220855/PMID: 32702298).

Data from vaccinated volunteers who received two vaccinations are included in this paper. Vaccine doses were either 5 × 10^10^ viral particles (standard dose; SD/SD cohort n = 21) or half dose as their first dose (low dose) and a standard dose as their second dose (LD/SD cohort n = 4). The interval between first and second dose was in the range of 8-14 weeks. Blood samples were collected and serum separated on the day of vaccination and on pre-specified days after vaccination e.g., 14 and 28 days after boost.

### Method details

#### COG-UK Sequence Analysis

COG-UK sequences from the 2nd February 2021 (Tatusov et al., 2000), and GISAID sequences (https://www.gisaid.org/) from South Africa from 30th January 2021 were downloaded and the protein sequence for the Spike protein was obtained after nucleotide 21000, followed by sequence alignment and recognition of mutations. The B.1.351 variant was filtered using selection criteria 501Y and Δ242. The B.1.1.7 variant was filtered using selection criteria 501Y and Δ69. The structural locations of mutations were modeled as red (single point mutations), black (deletions) or blue (additions) on the Spike structure with the size proportional to the logarithm of the incidence, and those mutations over 5% incidence in the population were explicitly labeled.

#### Focus Reduction Neutralization Assay (FRNT)

The neutralization potential of Ab was measured using a Focus Reduction Neutralization Test (FRNT), where the reduction in the number of the infected foci is compared to a negative control well without antibody. Briefly, serially diluted Ab or plasma was mixed with SARS-CoV-2 strain Victoria or B.1.351 and incubated for 1 hr at 37°C. The mixtures were then transferred to 96**-**well, cell culture-treated, flat-bottom microplates containing confluent Vero cell monolayers in duplicate and incubated for a further 2 hr followed by the addition of 1.5% semi-solid carboxymethyl cellulose (CMC) overlay medium to each well to limit virus diffusion. A focus forming assay was then performed by staining Vero cells with human anti-NP mAb (mAb206) followed by peroxidase-conjugated goat anti-human IgG (A0170; Sigma). Finally, the foci (infected cells) approximately 100 per well in the absence of antibodies, were visualized by adding TrueBlue Peroxidase Substrate. Virus-infected cell foci were counted on the classic AID EliSpot reader using AID ELISpot software. The percentage of focus reduction was calculated and IC_50_ was determined using the probit program from the SPSS package.

#### Cloning of native RBD, ACE2 and RBD K417N, E484K, N501Y

The constructs of native RBD and ACE2 are the same as in (Zhou et al., 2020). To clone RBD K417N, E484K, N501Y, a construct of RBD with the mutation N501Y (Supasa et al., 2021) was used as the template and four primers of RBD (K417N Forward primer 5′-CAGGGCAGACCGGCAATATCGCCGACTACAATTAC-3′, K417N reverse primer 5′-GTAATTGTAGTCGGCGATATTGCCGGTCTGCCCTG-3′, E484K Forward primer 5′-CACCGTGTAATGGCGTGAAGGGCTTCAATTGCTAC-3′ and E484K reverse primer 5′- GTAGCAATTGAAGCCCTTCACGCCATTACACGGTG-3′) and two primers of pNEO vector (Forward primer 5′- CAGCTCCTGGGCAACGTGCT-3′ and reverse primer 5′-CGTAAAAGGAGCAACATAG-3′) were used to do PCR. Amplified DNA fragments were digested with restriction enzymes AgeI and KpnI and then ligated with digested pNEO vector. This construct encodes exactly the same protein as native RBD except the K417N, E484K and N501Y mutations, as confirmed by sequencing.

#### Protein production

Protein production was as described in (Zhou et al., 2020). Briefly, plasmids encoding proteins were transiently expressed in HEK293T (ATCC CRL-11268) cells. The conditioned medium was dialysed and purified with a 5 mL HisTrap nickel column (GE Healthcare) and further polished using a Superdex 75 HiLoad 16/60 gel filtration column (GE Healthcare).

#### Bio-Layer Interferometry

BLI experiments were run on an Octet Red 96e machine (Fortebio). To measure the binding affinities of monoclonal antibodies and ACE2 with native RBD and RBD K417N, E484K, N501Y, each RBD was immobilized onto an AR2G biosensor (Fortebio). Monoclonal antibodies (Dejnirattisai et al., 2021) were used as analytes or serial dilutions of ACE2 were used as analytes. All experiments were run at 30°C. Data were recorded using software Data Acquisition 11.1 (Fortebio) and Data Analysis HT 11.1 (Fortebio) with a 1:1 fitting model used for the analysis.

### Quantification and statistical analysis

Statistical analyses are reported in the results and figure legends. Neutralization was measured by FRNT. The percentage of focus reduction was calculated and IC_50_ was determined using the probit program from the SPSS package.The Wilcoxon matched-pairs signed rank test was used for the analysis and two-tailed P values were calculated and geometric mean values. BLI data were analyzed using Data Analysis HT 11.1 (Fortebio) with a 1:1 fitting model.
