# Supplementary material for: Evidence of escape of SARS-CoV-2 variant B.1.351 from natural and vaccine-induced sera
Source: Cell. 2021 Apr 29;184(9):2348–2361.e6. doi: 10.1016/j.cell.2021.02.037 (PMC7901269; doi:10.1016/j.cell.2021.02.037)
Supplement: Document S1. Tables S1–S3 [file mmc1.pdf]

Cell, Volume *189*

Supplemental information

Evidence of escape of SARS-CoV-2 variant B.1.351 from natural and vaccine-induced sera

Daming Zhou, Wanwisa Dejnirattisai, Piyada Supasa, Chang Liu, Alexander J. Mentzer, Helen M. Ginn, Yuguang Zhao, Helen M.E. Duyvesteyn, Aekkachai Tuekprakhon, Rungtiwa Nutalai, Beibei Wang, Guido C. Paesen, Cesar Lopez-Camacho, Jose Slon-Campos, Bassam Hallis, Naomi Coombes, Kevin Bewley, Sue Charlton, Thomas S. Walter, Donal Skelly, Sheila F. Lumley, Christina Dold, Robert Levin, Tao Dong, Andrew J. Pollard, Julian C. Knight, Derrick Crook, Teresa Lambe, Elizabeth Clutterbuck, Sagida Bibi, Amy Flaxman, Mustapha Bittaye, Sandra Belij-Rammerstorfer, Sarah Gilbert, William James, Miles W. Carroll, Paul Klenerman, Eleanor Barnes, Susanna J. Dunachie, Elizabeth E. Fry, Juthathip Mongkolsapaya, Jingshan Ren, David I. Stuart, and Gavin R. Screaton

Table S1A

Convalescent plasma	FRNT50 (Recprocal plasma dilution)		Victoria/B.1.351 ratio
	Victoria	B.1.351	
Convalescent 1	61	<20	>3.1
Convalescent 2	689	<20	>34.4
Convalescent 3	526	88	6.0
Convalescent 4	409	339	1.2
Convalescent 5	369	<20	>18.4
Convalescent 6	1270	522	2.4
Convalescent 7	274	<20	>13.7
Convalescent 8	633	111	5.7
Convalescent 9	667	<20	33.3
Convalescent 10	124	<20	>6.2
Convalescent 11	102	<20	>5.1
Convalescent 12	339	<20	>16.9
Convalescent 13	331	<20	>16.6
Convalescent 14	438	<20	>21.9
Convalescent 15	6397	1147	5.6
Convalescent 16	44	<20	>2.2
Convalescent 17	1115	<20	>55.8
Convalescent 18	242	<20	>12.1
Convalescent 19	29	<20	>1.5
Convalescent 20	154	21	7.4
Convalescent 21	487	58	8.5
Convalescent 22	438	53	8.2
Convalescent 23	381	<20	>19.1
Convalescent 24	1647	72	22.9
Convalescent 25	913	202	4.5
Convalescent 26	1880	348	5.4
Convalescent 27	1464	67	21.9
Convalescent 28	361	<20	>18.0
Convalescent 29	2859	748	3.8
Convalescent 30	1109	184	6.0
Convalescent 31	811	210	3.9
Convalescent 32	395	<20	>19.8
Convalescent 33	1144	48	23.7
Convalescent 34	676	<20	>33.8

Table S1B.

Kent plasma	Day after admission	FRNT50 (Recprocal plasma dilution)		Victoria/B.1.351 ratio
		Victoria	B.1.351	
B.1.1.7 P1	5	<20	<20	N/A
B.1.1.7 P2	3	<20	<20	N/A
B.1.1.7 P3	1	440	<20	>22
B.1.1.7 P4	1	136884	81493	1.7
B.1.1.7 P5	29	1506	278	5.4
B.1.1.7 P6	24	370	80	4.6
B.1.1.7 P7	25	2250	159	14.2
B.1.1.7 P8	18	2999	867	3.5
B.1.1.7 P9	20	970	336	2.9
B.1.1.7 P10	14	3735	1150	3.2
B.1.1.7 P11	18	2193	705	3.1
B.1.1.7 P12	29	<20	<20	N/A
B.1.1.7 P13	34	<20	<20	N/A

Table S1. FRNT50 titres against Victoria and B.1.351 strains (A) 34 convalescent plasma (B) Plasma from 13 patients infected with B.1.1.7.The data underpinning the Victoria neutralization curves have been previously reported (Supasa et al, 2021). , related to STAR Methods Focus Reduction Neutralisation Assay (FRNT) and Figure 2.

Table S2

Vaccine samples	Day Post-boost	FRNT50 (Recprocal plasma dilution)		Victoria/B.1.351 ratio
		Victoria	B.1.351	
Pfizer1	7	1149	73	15.7
Pfizer2	7	<20	<20	N/A
Pfizer3	7	1727	230	7.5
Pfizer4	8	2234	420	5.3
Pfizer5	7	3016	577	5.2
Pfizer6	7	1521	152	10.0
Pfizer7	7	609	109	5.6
Pfizer8	7	4340	1255	3.5
Pfizer9	7	1467	102	14.4
Pfizer10	7	1757	124	14.2
Pfizer11	7	860	121	7.1
Pfizer12	7	1749	66	26.6
Pfizer13	7	1851	385	4.8
Pfizer14	7	407	122	3.3
Pfizer15	8	1285	202	6.4
Pfizer16	8	1286	91	14.1
Pfizer17	8	1810	143	12.7
Pfizer18	8	1198	93	12.9
Pfizer19	8	466	61	7.6
Pfizer20	8	1539	178	8.7
Pfizer21	9	184	<20	>9.2
Pfizer22	11	1061	212	5.0
Pfizer23	12	1658	100	16.6
Pfizer24	12	1155	192	6.0
Pfizer25	15	8092	3006	2.7
AstraZeneca 1	28	495	155	3.2
AstraZeneca 2	28	580	217	2.7
AstraZeneca 3	28	253	<20	>12.6
AstraZeneca 4	28	183	62	3.0
AstraZeneca 5	28	432	62	7.0
AstraZeneca 6	28	764	54	14.3
AstraZeneca 7	28	133	<20	>6.7
AstraZeneca 8	28	257	41	6.3
AstraZeneca 9	28	501	128	3.9
AstraZeneca 10	28	357	116	3.1
AstraZeneca 11	14	334	45	7.5
AstraZeneca 12	14	250	51	4.9
AstraZeneca 13	14	122	<20	>6.1
AstraZeneca 14	14	212	<20	>10.6
AstraZeneca 15	14	789	94	8.4
AstraZeneca 16	14	538	57	9.5
AstraZeneca 17	14	1159	36	32.5
AstraZeneca 18	14	353	44	8.1
AstraZeneca 19	14	975	69	14.0
AstraZeneca 20	14	169	<20	>8.4
AstraZeneca 21	14	155	<20	>7.7
AstraZeneca 22	14	152	<20	>7.6
AstraZeneca 23	14	126	<20	>6.3
AstraZeneca 24	14	293	30	9.7
AstraZeneca 25	14	94	<20	>4.7

Table S2. FRNT50 titres against Victoria and B.1.351 strains (A) Serum from 25 recipients of Pfizer-BioNTech vaccine. (B) Oxford-AstraZeneca vaccine. The data underpinning the Victoria neutralization curves have been previously reported (Supasa et al, 2021). Related to Figure 3 and STAR Methods Focus Reduction Neutralisation Assay (FRNT).

Table S3A

mAb	IC50 (ug/ml)		B.1.351/Victoria	KD (nM)		Immunoglobulin gene usage		
	Victoria	B.1.351	ratio	Native RBD	RBD B.1.351	IGHV	K/λ	IGLV
40	0.026 ± 0.007	0.738 ± 0.311	28.6	0.55±0.003	33.2±0.88	3-66	K	1-33 or 1D-33
55	0.095 ± 0.015	0.127 ± 0.014	1.3	3.61±0.15	17.6±0.57	1-58	K	3-20
58	0.041 ± 0.003	0.136 ± 0.010	3.3	0.30±0.003	5.96±0.12	3-9	λ	3-21
88	0.033 ± 0.001	>10	>615.3	0.64±0.004	277.3±7.17	4-61	λ	1-36
132	0.048 ± 0.000	>10	>416.8	16.0±0.09	No signal	4-34	λ	7-46
150	0.012 ± 0.000	0.350 ± 0.010	30.0	0.19±0.002	227.7±8.58	3-53	K	1-9
158	0.031 ± 0.004	>10	>654.9	0.53±0.0003	976.5±30.7	3-53	K	1-9
159	0.011 ± 0.000	>10	>1856.8	N/A	N/A	3-30	K	3-20
165	0.034 ± 0.004	0.054 ± 0.013	1.6	3.49±0.15	8.57±0.20	1-58	K	3-20
170	0.025 ± 0.004	>10	>804.4	5.75±0.07	1207±74.94	5-51	K	2D-29
175	0.026 ± 0.000	>10	>783	0.97±0.005	1350±45.9	3-53	K	1-33 or 1D-33
222	0.019 ± 0.000	0.017 ± 0.005	0.9	0.29±0.003	0.47±0.09	3-53	K	3-20
253	0.055 ± 0.008	0.109 ± 0.055	2.0	13.9±0.48	12.1±0.25	1-58	K	3-20
269	0.030 ± 0.000	>10	>675	1.59±0.009	>1000	3-53	K	1-9
278	0.014 ± 0.007	0.160 ± 0.018	11.7	5.11±0.03	8.84±0.16	1-18	K	1-39 or 1D-39
281	0.005 ± 0.001	>10	>4052.7	3.20±0.01	79540±170	3-7	K	2-24
316	0.018 ± 0.007	>10	>1127.2	1.50±0.01	No signal	1-2	λ	2-8
318	0.029 ± 0.008	0.019 ± 0.008	0.7	2.23±0.02	3.26±0.04	1-58	K	3-20
384	0.004 ± 0.001	>10	>4796.9	0.51±0.004	No signal	3-11	K	1-27
398	0.091 ± 0.004	>10	>220.5	1.02±0.007	21.4±0.20	3-66	λ	2-8
253-55	0.003 ± 0.000	0.009 ± 0.002	2.7	0.88±0.02	3.43±0.06	1-58	3-20	3-20
253-165	0.003 ± 0.000	0.013 ± 0.003	3.6	0.73±0.01	3.10±0.05	1-58	3-20	3-20

Table S3B

mAb	IC50 (ug/ml)		B.1.351/Victoria	KD (nM)	
	Victoria	B.1.351	ratio	Native RBD	RBD B.1.351
REGN R10987	0.032 ± 0.007	0.007 ± 0.001	0.2	3.85±0.08	0.72±0.02
REGN R10933	0.004 ± 0.002	3.284 ± 2.014	773.7	1.82±0.03	No signal
AZD1061	0.013 ± 0.003	0.014 ± 0.002	1.1	1.04±0.01	1.52±0.02
AZD8895	0.005 ± 0.001	0.046 ± 0.031	8.9	1.49±0.02	3.63±0.06

Supplementary Table 3. FRNT50 titres against Victoria and B.1.351 strains (A) 22 human monoclonal antibodies. (B) Two Regeneron and 2 AstraZenca monoclonal antibodies. The Mann–Whitney U test was used for the analysis and two-tailed P values were calculated, data are shown as mean ± s.e.m. The data underpinning the Victoria neutralization curves have been previously reported (Supasa et al, 2021). Related to Figure 4 and STAR Methods Focus Reduction Neutralisation Assay (FRNT).
